# Decoding Functional and Developmental Trajectories of Tissue-Resident Uterine Dendritic Cells Through Integrative Omics

**DOI:** 10.21203/rs.3.rs-5424920/v1

**Published:** 2024-11-14

**Authors:** Gil Mor, Aditi Singh, Jing Yang, Nicholas Adzibolosu, Songchen Cai, Elana Kauf, Lingtao Yang, Qiyuan Li, Hanjie Li, Alexandra Werner, Siddharth Parthasarathy, Jiahui Ding, Jared Fortier, Marta Rodriguez- Garcia, Liang-Hui Diao

**Affiliations:** Wayne State University; Wayne State University; National Institute for Data Science in Health and Medicine, School of Medicine, Xiamen University, Xiamen 361102, China; Wayne State University; Shenzhen Zhongshan Obstetrics & Gynecology Hospital; Wayne State University-; Shenzhen Zhongshan; National Institute for Data Science in Health and Medicine, School of Medicine, Xiamen University, Xiamen 361102, China; Shenzhen Institutes of Advanced Technology; Wayne State University; Tufts University; Wayne State University school of Medicine; Wayne State University; Wayne State University; Sun Yat-sen University

## Abstract

Uterine dendritic cells (uDCs) are critical for endometrial function, yet their origin, molecular characteristics, and specific roles during the pre- and post-implantation periods in the human endometrium remain largely unknown. The complexity of the endometrial environment makes defining the contributions of uDCs subtypes challenging. We hypothesize that distinct uDC subsets carry out specialized functions, and that resident progenitor DCs generate these subtypes. Employing single-cell RNA sequencing on uterine tissues collected across different menstrual phases and during early pregnancy, we identify several uDCs subtypes, including resident progenitor DCs. CITE-seq was performed on endometrial single-cell suspensions to link surface protein expression with key genes identified by the RNAseq analysis. Our analysis revealed the developmental trajectory of the uDCs along with the distinct functional roles of each uDC subtype, including immune regulation, antigen presentation, and creating a conducive environment for embryo implantation. This study provides a comprehensive characterization of uDCs, serving as a foundational reference for future studies for better understanding female reproductive disorders such as infertility and pregnancy complications.

## Introduction

Reproductive medicine has made significant progress, yet the underlying mechanisms that control embryo implantation and pregnancy remain poorly understood. One critical component of this process is the human endometrium, the dynamic inner lining of the uterus which undergoes dramatic changes throughout the menstrual cycle and, in the absence of pregnancy, completely remodels every month. The menstrual cycle, (1) averages 28 days and is divided into two major phases by the event of ovulation (day 14): proliferative phase, before ovulation(2). During the proliferative phase, the endometrium regenerates, while the secretory phase involves differentiation and preparation for pregnancy. During the secretory phase the endometrium enters a narrow period of cellular and molecular receptivity, ideal for embryo implantation, known as the window of implantation (days 19–23 of the menstrual cycle). Without pregnancy, the endometrium sheds, initiating a new cycle of regeneration(3). The source of the different cell types associated with each menstrual remodeling remains poorly understood.

Immune cells, including B cells, T cells, macrophages, Natural Killer (NK) cells and Dendritic cells (DCs), are major components of the human endometrium (4, 5). Some immune cells form lymphoid aggregates, with a B cell core surrounded by CD8 + T cells and encircled by macrophages (6). Recent studies show the expansion of CD8 + tissue-resident memory (TRM) cells, innate lymphoid cells (ILC1), and NK cells, during subsequent pregnancies, indicating their stable persistence in the basal layer between pregnancies (7) (8) (9). The origin of other immune cells such as DCs is unknown.

DCs are specialized antigen-presenting cells crucial for capturing, processing, and presenting antigens to T cells, thus initiating and regulating immune responses (10). DCs include conventional dendritic cells (cDCs), plasmacytoid dendritic cells (pDCs), and monocyte-derived dendritic cells (moDCs) (11), each with distinct functions and localizations. DCs are crucial for activating naive T cells (12, 13), initiating adaptive immune responses and maintaining tolerance by presenting self-antigens in a non-inflammatory context, preventing autoimmunity and maintaining tissue homeostasis (14).

In the human endometrium, DCs balance tolerance to a semi allogeneic fetus and defense against pathogens during implantation (15, 16). DCs and macrophages create an inflammatory gradient affecting the mucin layer on the epithelium, which allows the apposition and adhesion of the blastocyst to the epithelium and promotes implantation (17, 18). We previously showed that depleting uDCs severely impairs implantation and leads to embryo resorption (17). This impairment is related not to immune tolerance, but to successful decidualization (19, 20). These findings indicate that, besides their immune functions, uDCs also have a trophic role in regulating implantation. Despite their recognized importance, the origin, molecular characteristics, and precise roles of uDCs in the endometrium remain unknown. The complex endometrial milieu, cellular heterogeneity, and dynamic molecular signaling pose significant challenges in understanding the specific contributions of uDC subtypes.

We hypothesized that the diverse functions of DCs in the endometrium are fulfilled by different subsets of DCs, with renewal provided by tissue resident progenitor cells. To test this hypothesis, we performed single cells analysis of uDCs throughout the menstrual cycle and early pregnancy. Our data reveal, for the first time, the existence of multiple sub-types of DCs in the human endometrium. More importantly, we show the presence of resident progenitor uDCs within the endometrium, suggesting a self-renewing source of these pivotal cells. These findings challenge the notion that DCs are solely recruited from peripheral blood, offering fresh perspectives on the immunological underpinnings of endometrial receptivity and blastocyst implantation.

## Results

### 1. Identification of multiple unique populations of endometrial DCs

**Figure 1:**
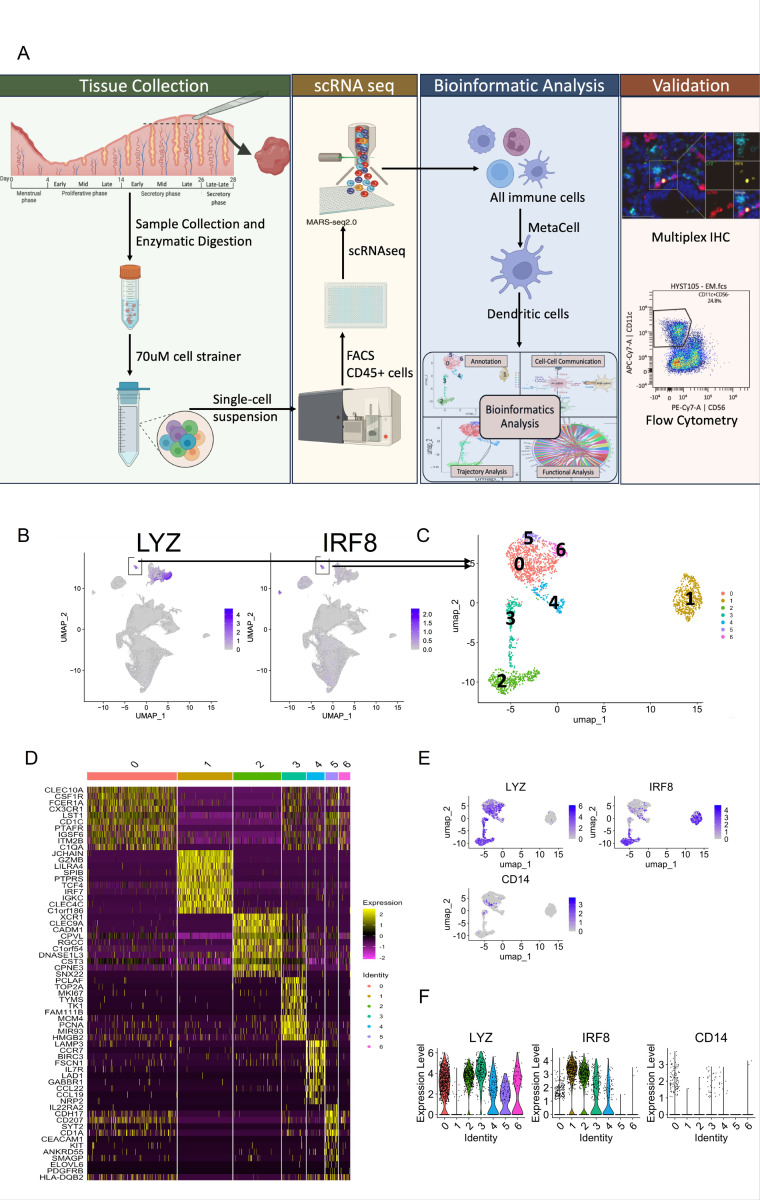
Comprehensive analysis of uterine dendritic cell (uDC) subsets and their gene expression profiles. **(A)** Schematic representation of the sample collection process and subsequent single-cell RNA sequencing, CITE-seq and multiplex IHC workflow. Uterine tissue samples were enzymatically digested to obtain single-cell suspensions, which were then sorted via FACS to isolate CD45+ immune cells. The isolated cells underwent scRNA-seq analysis using the involving MARS-seq2.0 protocol. Subsequent bioinformatic analysis enabled the identification and characterization of distinct dendritic cell subpopulations. The findings were further validated through wet-lab techniques, including flow cytometry and immunohistochemistry (IHC), to confirm the presence of these dendritic cell subpopulations. **(B)** UMAP plot showing all immune cells in Grey and cells expressing LYZ and IRF8 in blue. Small subsets (indicated in boxes) were identified as Dendritic Cells which was selected and used for downstream analysis. **(C)** UMAP plot visualizing the clustering of uDCs based on their gene expression profiles. Each point represents a single cell, colored according to the identified cluster (0–6), illustrating the distinct transcriptional landscapes within the uDC population. **(D)** Heatmap showing the expression of Top 10 marker genes (shown on y-axis) across different uDC clusters (shown on x-axis). Each row represents a gene, and each column corresponds to a single cell, organized by cluster identity. Yellow color denotes upregulated genes whereas pink denotes expression of the downregulated genes. **(E)** Spatial distribution for key markers (LYZ, IRF8, and CD14) across the uDC clusters shown on UMAP. **(F)** Expression level for key marker genes (LYZ, IRF8, and CD14) was quantified using Violin plots. X-axis shows the clusters and y-axis shows the expression level of the genes indicated on top of each plot.

To characterize the uDC population present in the human endometrium throughout the menstrual cycle, we performed Single-cell RNA sequencing (scRNAseq) analysis and determined the transcriptomic landscape of uDCs using LYZ + and IRF8 + markers. A total of 2229 dendritic cells were extracted from uterine samples across different menstrual cycle stages: Proliferative (Early, mid, and late); Secretory (Early, mid, and late); Menstrual; and early pregnancy (decidua basalis and decidua parietalis) (Fig. 1A and 1B).

The analysis of *LYZ*+/ *IRF8 +* DCs revealed a rich diversity within the uDCs population, identifying seven distinct clusters depicted in a heatmap, with the top 10 genes for each cluster (Fig. 1C). The heatmap illustrates unique gene expression profiles for clusters 1 through 5, while clusters 0 and 6 demonstrate overlapping gene expression patterns, suggesting a shared transcriptional signature between these two subtypes of uDCs (Fig. 1C).

To better identify the different subpopulations of uDC, we used Uniform Manifold Approximation and Projection (UMAP) analysis revealing seven distinct subtypes of uDCs (Fig. 1D). This unbiased clustering analysis did not rely on known DC markers. UMAP highlighted the complexity and heterogeneity within the uDC population, indicating multiple distinct transcriptomic programs within what was previously considered a singular cell type.

We confirmed the identified populations as DCs, by assessing *LYZ* and *IRF8* expression on the UMAP. Interestingly, all the clusters, except for cluster 1, were positive for *LYZ* (Fig. 1E) while cluster 1 was positive for *IRF8*. Only Clusters 2 and 3 were double positive for *LYZ*^*+*^*/IRF8*^+^(Fig. 1E). Violin plots illustrate the expression level of these genes (Fig. 1F), and none of the clusters showed *CD14 +* cells (monocyte/macrophage marker), confirming the specificity of the approach for classical DCs (Fig. 1F).

### 2. Characterization of peripheral blood derived uDCs (PB-uDC)

**Figure 2:**
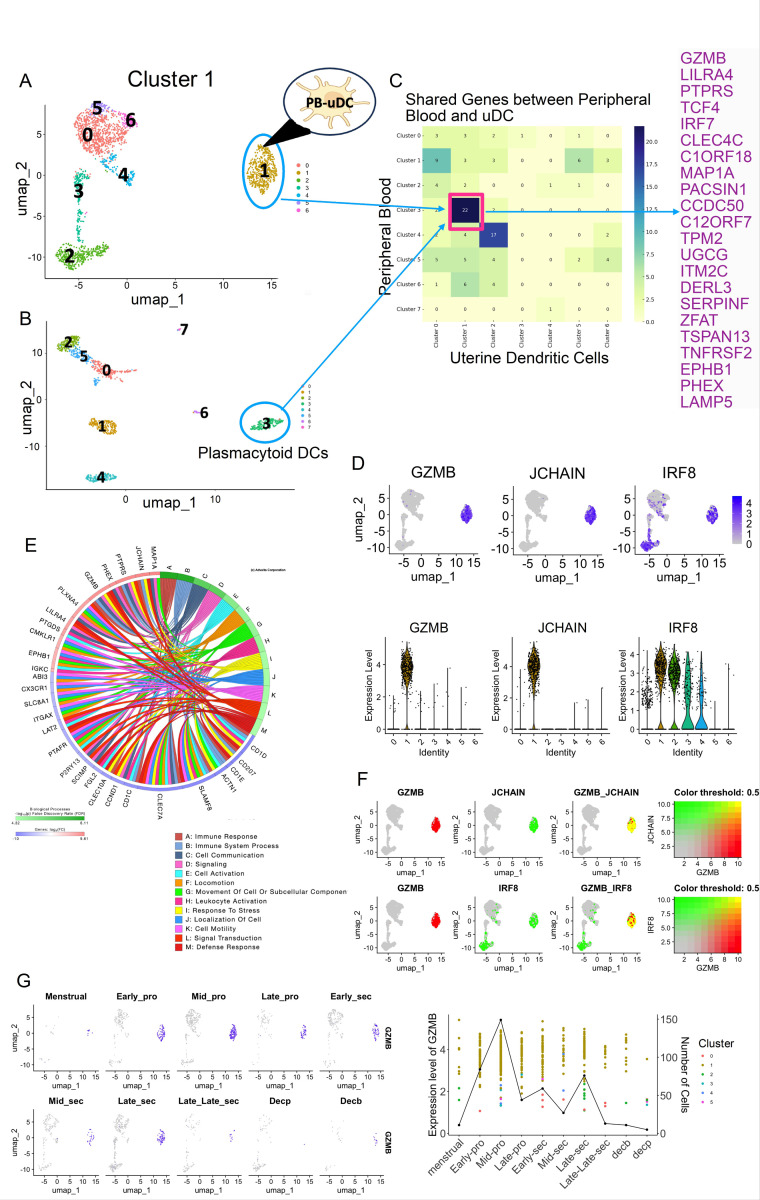
Comparative analysis of PBMC DC and uterine DCs. **(A)** UMAP visualization of DC from the uterine tissues showing 7 clusters and cluster 1 identified as PB-uDCs. **(B)** UMAP visualization of DC from the PBMC dataset showing 8 clusters, with cluster 3 highlighted which was identified as plasmacytoid DCs. **(C)** Heatmap showing number of genes shared from comparing the Top 50 markers from clusters 0–7 from PBMC and clusters 0–6 from uDCs. Cluster 1 from uDC has the highest number of shared genes with cluster 3 of PBMC DCs is highlighted in black and the list of 22 shared genes is shown on the side. The number of genes shared with each cluster of uDCs with each cluster of Peripheral Blood DCs are shown in the box. **(D)** UMAP showing expressions of marker genes such as GZMB, JCHAIN and IRF8 within Cluster 1. Violin plots below show the quantification of expression levels of these marker genes in different clusters. GZMB and JCHAIN were specifically expressed in cluster 1, whereas IRF8 was found in clusters 1, 2, 3 and 4.**(E)** Circos plots showing the Biological Processes for the differentially expressed genes in the PB-uDC cluster along with the top 10 genes associated with each pathway. Red color indicates the genes were up-regulated; blue color indicates that the genes were down-regulated. **(F)** UMAP showing co-expression of the marker genes of the cluster- GZMB, JCHAIN and IRF8. Yellow color indicates genes are co-expressed. **(G)** UMAP showing the expression of the marker gene GZMB across 10 phases of the menstrual cycle, from the Menstrual stage through to Decidualization (Decp and Decb), indicating dynamic changes in gene expression. Alongside is shown **a l**ine graph combined with a scatter plot overlay depicting the expression level of GZMB marker gene (1^st^ y-axis) and number of cells expressing the gene (2^nd^ y-axis) across different stages of the menstrual cycle (X-axis). Each point represents the expression level of a gene in a particular cluster, while the line graph emphasizes the overall expression trend, aiding in the identification of patterns or anomalies in gene expression related to specific menstrual phases.

The existing dogma is that DCs are recruited from the peripheral blood to the uterus (21). From the cluster analysis, we observed that Cluster 1 is the most diverse cluster in terms of gene signature and markers expression (*LYZ*^*−*^*/IRF8*^*+*^) (Fig. 1D); therefore, we hypothesized that cluster 1 might have been recruited from peripheral blood while the rest of the clusters are tissue resident DCs. To test this hypothesis, we compared the newly identified clusters of uDCs (Fig. 2A) to DC subpopulations present in PBMCs using the publicly available scRNAseq data (Fig. 2B and (22)). Our UMAP analysis delineated eight distinct clusters among the PBMC DCs (Fig. 2B). We then performed a Meta analysis for the transcriptome of PB-uDC and peripheral blood DC clusters and found greatest gene overlap between PB-uDC and cluster 3 of the peripheral blood DCs (Fig. 2A–B), which has been described as plasmacytoid DCs (22). We compared the Top 50 genes from Cluster 1 uDCs to cluster 3 peripheral blood DCs and found 22 genes to be shared (Fig. 2C), while similar number of genes are unique for each cell type. These findings suggest that upon migrating into the uterus, Cluster 1 undergoes differentiation acquiring unique characteristics corresponding to uDCs. Based on these findings we referred Cluster 1 as PB-uDC.

Next, we defined the functional characteristics of this cluster. So, we extracted all differentially expressed p-value significant genes (P < 0.05), and log fold change of at least 0.6, for the PB-uDC sub-type and used iPathway analysis. The biological processes that were found include immune response, cell communication, signaling, cell activation, locomotion, leukocyte activation, response to stress, localization, motility, signal transduction and defense response, with the genes associated with each process (Fig. 2E). To determine specific markers and characteristics of PB-uDCs, we identified DC-genes, *GZMB* and *JCHAIN* from the bubble plot (Supp. Figure 1). We observed that *GZMB* and *JCHAIN* are only expressed in PB-uDCs (Fig. 2D); and co-expression of *GZMB* and *JCHAIN* is only detected in PB-uDCs (Fig. 2F). *IRF8* is detected in PB-uDCs as well as in clusters 2 and 3; however, the co-expression of *GZMB* and *IRF8* is found only in PB-uDCs (Fig. 2F). Violin plots confirmed that *JCHAIN* and *GZMB* were specific to PB-uDCs (cluster 1) whereas *IRF8* was found in clusters 1, 2, 3 and 4 as well (Fig. 2F).

Finally, we evaluated the presence of PB-uDCs during different stages of the menstrual cycle. PB-uDCs were nearly undetectable in the endometrial samples collected at menstruation (Fig. 2G). Their numbers increase at early proliferative, peak at mid-proliferative, decrease afterwards (Fig. 2G). PB-uDCs are not detected in early decidua parietalis nor basalis (Fig. 2G). This finding supports our hypothesis that cluster 1 represents a subset of uDC recruited from peripheral blood in contrast with the other clusters which might have differentiated from local precursor cells.

### 3. Identification of Resident/progenitor uDCs (P-uDCs)

**Figure 3:**
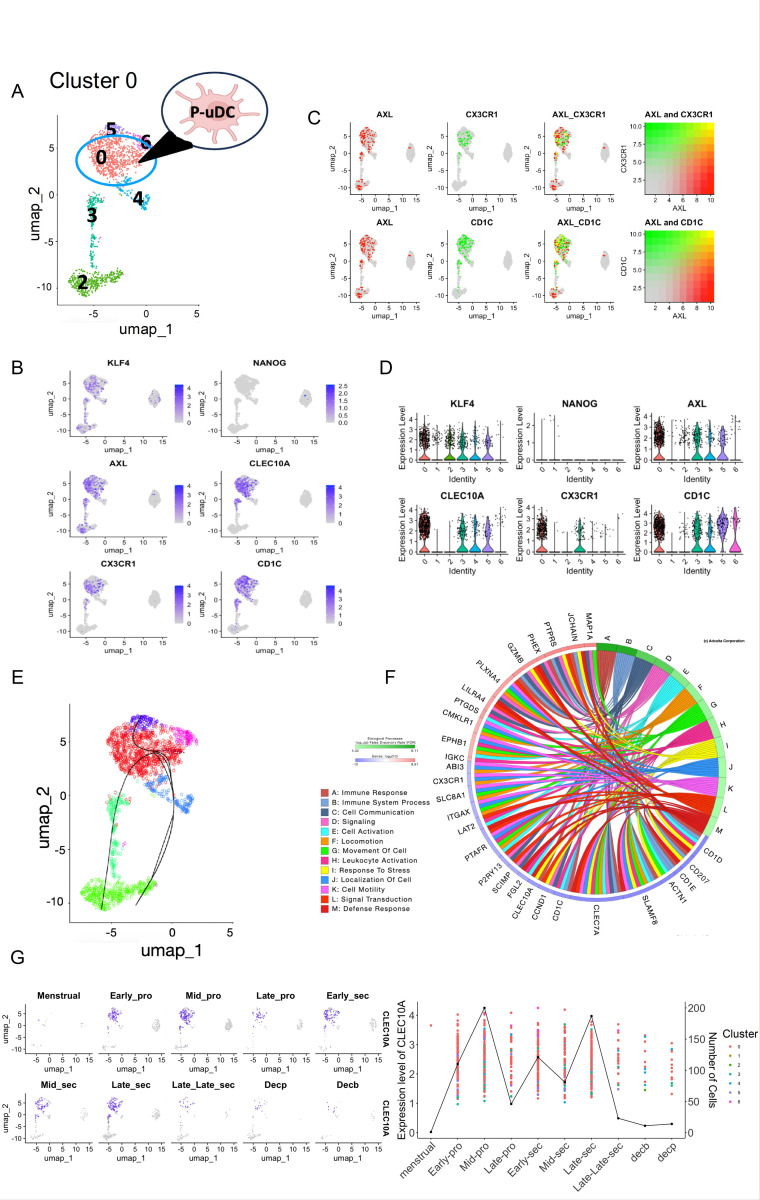
Identification and characterization of progenitor cells on the UMAP. **(A)** UMAP visualization uDCs with cluster 0 being identified as progenitor uDCs. **(B)** UMAP plot depicts the expression of key marker genes for stem cells, including KLF4 and NANOG, as well as known marker genes for DC progenitors, such as AXL, CLEC10A, CX3CR1, and CD1C. In the plot, all cells are represented in gray, while cells expressing the genes listed at the top of the UMAP are highlighted in purple. **(C)** UMAP Highlighting co-expression patterns of DC progenitor marker genes. This panel illustrates cells co-expressing progenitor marker genes AXL and CX3CR1, as well as cells co-expressing AXL and CD1C, within the UMAP. **(D)** Violin plots showing quantification for stem cell marker genes and DC progenitor marker genes are shown. Y-axis shows the expression level and x-axis shows the clusters. **(E)** Lineage tracing using predictive slingshot indicates cluster 0 is progenitor from which all other clusters originate. The lines originate from Cluster 0 and go through cluster 3 to cluster 2, another line originating from cluster 0 goes directly to clusters 4 and 5. **(F)** Circos plots showing the Biological processes found for the genes differentially expressed in the P-uDC cluster along with the Top 10 genes associated with each pathway. The genes are color coded red for up-regulation and blue for down-regulation. **(G)** UMAP showing the expression of the marker gene CLEC10A across 10 phases of the menstrual cycle, from the Menstrual stage through to Decidualization (Decp and Decb), indicating dynamic changes in gene expression. Alongside is a line graph combined with a scatter plot overlay depicting the expression level of CLEC10A marker genes (1^st^ y-axis) and number of cells expressing the gene (2^nd^ y-axis) across different stages of the menstrual cycle (x-axis). Each point represents the expression level of a gene in a particular cluster, while the line graph emphasizes the overall expression trend.

While cluster 1 (PB-uDCs) showed major separation in the UMAP, the rest of the clusters, although different, grouped closer together (Fig. 1C). Consequently, we hypothesized that the remaining clusters may represent tissue resident uDCs and have their origin from precursor cells located within the endometrium (Fig. 3A). To test this hypothesis, we sought to ascertain a cluster with characteristics of progenitor cells by looking at the expression of *KLF4*, *NANOG*, *AXL*, *CX3CR1*, *CD1C*, and *CLEC10A*.

*CX3CR1*, and *CLEC10A* were identified as specific markers for cluster 0 by bubble plot (Supp. Figure 1)

*KLF4* and *AXL* were found expressed in all the clusters but PB-uDCs (cluster 1), confirming a common origin for the rest of the clusters (Fig. 3B). *CX3CR1*, CLEC10A and *CD1C* were localized only in clusters 0 and 3 but not in clusters 4,5 and 2, signifying that either clusters 0 or 3 are the tissue progenitor uDCs (Fig. 3B). We observed that *CX3CR1* is mainly expressed in cluster 0 and few cells also expressing *CX3CR1* in cluster 3 (Fig. 3B). *CLEC10A* and *CD1C* are mostly expressed in cluster 0 but also in a sub-group of clusters 3 (Fig. 3B). *AXL* is expressed on all the clusters but cluster 1, suggesting *AXL* as a common marker for tissue residents uDCs (Fig. 3B). *AXL* was found to be co-expressed with *CX3CR1* and *CD1C* in cluster 0 and slightly in cluster 3 (Fig. 3C). Violin plots validate that *CX3CR1, CLEC10A* and *CD1C* were specifically found mainly in cluster 0 (Fig. 3D). *NANOG* was not found expressed in any of the clusters (Fig. 3B and D).

To determine which of the two clusters may represent the tissue progenitor uDCs, we utilized the Slingshot algorithm for lineage tracing and observed a developmental trajectory from cluster 0 through cluster 3 to cluster 2 and to clusters 5, 6 and 4 (Fig. 3E). This path supports the hypothesis that cluster 0 indeed serves as a progenitor state giving rise to other differentiated DC populations.

Functional characteristics of this progenitor cluster were analyzed by examining the 784 differentially expressed genes (DEGs) that were statistically significant (p-value < 0.05), and log fold change of at least 0.6, using iPathway analysis. (23, 24), (25). The functional characteristics of this cluster, depicted by the gene regulatory networks and signaling pathways, are associated with regulation of inflammation and tolerance, involving complement and Type I and II interferon (Fig. 3F). Related to the process of tolerance (allograft rejection) we identified *IL-1b, FCGR2b, IGSF6, CD1D, IL-18* and *CTSH*, highly expressed in this cluster. These genes have been identified as immune regulatory factors modulating inflammation, immune cell activation and antigen presentation (26–30).

Using *CLEC10A* as a specific marker for P-uDCs, we evaluated their presence during the different stages of the menstrual cycle. P-uDCs numbers increase during the mid-proliferative and late secretory phases of the menstrual cycle (Fig. 3G). These findings define cluster 0 as the potential tissue resident progenitor uDCs (P-uDC).

### 4. Transitional/Proliferative uDCs (T-uDCs) were found in cluster 3 of the UMAP

**Figure 4:**
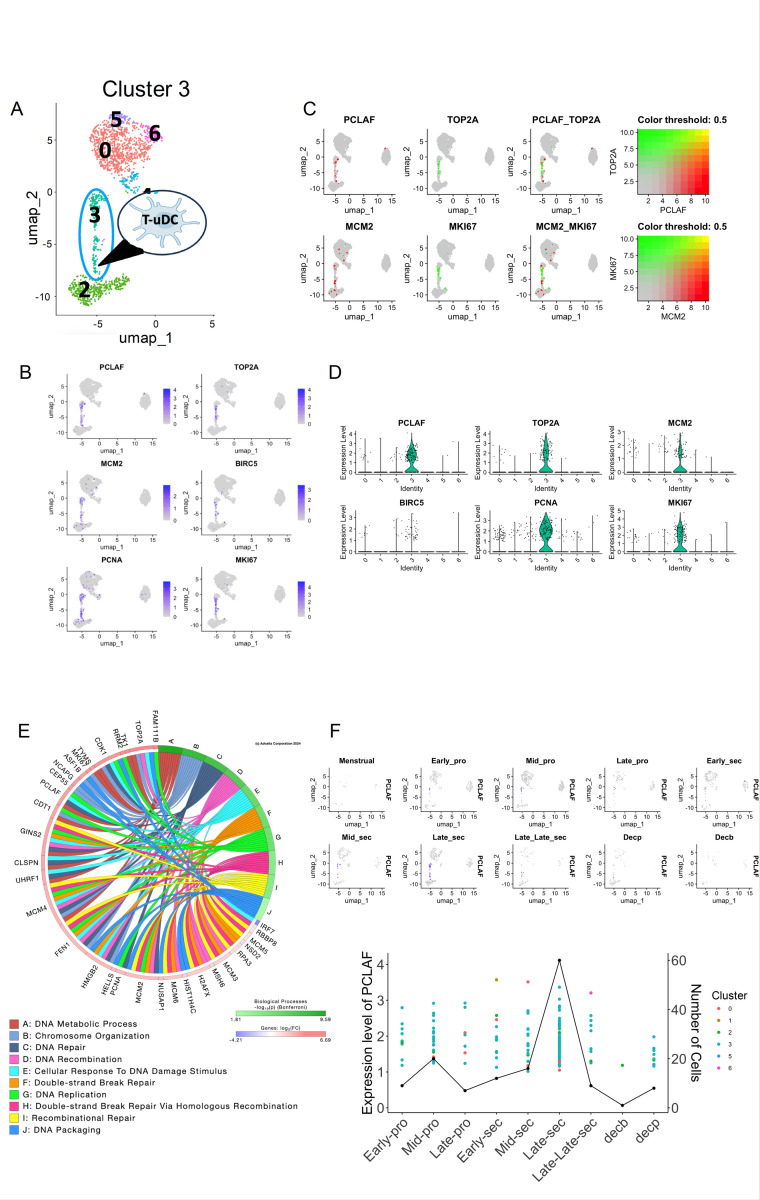
Identification and characterization of Proliferating/transitional uDCs (T-uDCs). **(A)** UMAP visualization uDCs with cluster 3 being identified as transitional uDCs. **(B)** UMAP plot depicts the expression of key marker genes for proliferating cells, including PCLAF, TOP2A, PCNA, BIRC5, MCM2, MKI67. In the plot, all cells are represented in gray, while cells expressing the genes listed at the top of the UMAP are highlighted in purple. **(C)** Highlighting co-expression patterns of proliferative marker genes. This panel illustrates cells co-expressing progenitor marker genes PCLAF and TOP2A, as well as cells co-expressing MCM2 and MKI67, within the UMAP. **(D)** Violin plots for proliferation marker genes are shown. Y-axis shows the expression level and x-axis shows the clusters. **(E)** Circos plots showing the biological processes for the T-uDC cluster along with the genes associated with each pathway. The genes are color coded red for up-regulation and blue for down-regulation. Processes are mostly cell-cycle related such as DNA metabolic process, chromosome organization, DNA repair, recombination, replication and packaging. **(F)** UMAP showing the expression of the marker gene PCLAF across 10 phases of the menstrual cycle, from the Menstrual stage through to Decidualization (Decp and Decb), indicating dynamic changes in gene expression. Below is shown a line graph combined with a scatter plot overlay depicting the expression level of PCLAF (1^st^ y-axis) and number of cells expressing the gene (2^nd^ y-axis) across different stages of the menstrual cycle (x-axis). Each point represents the expression level of a gene in a particular cluster, while the line graph emphasizes the overall expression trend.

Since we observed multiple similarities between cluster 0 (P-uDCs) and cluster 3, we hypothesized that cluster 3 may represent a transitional stage of DC differentiation (Fig. 4A). To test this, we analyzed specific genes for this cluster using Bubble Plot and identified *PCLAF, TOP2A, MKI67, TYMS* and *TK1* (Supp. Figure 1). Interestingly, these genes are associated with cell proliferation (31–34) and were found to be expressed only in cluster 3 (Fig. 4B). The co-expression and expression levels across different cells within the cluster were quantified and visualized using violin plots, demonstrating significant expression of these proliferative markers (Fig. 4C–D).

To understand the function of the cluster, we extracted the 1000 DEGs that were statistically significant (p-value < 0.05) and had a log fold change of at least 0.6. The functional analysis of this cluster was characterized by an abundance of cell cycle-associated pathways, including E2F targets, MYC targets, and G2/M checkpoints (Fig. 4E). This enrichment in pathways critical for cell proliferation confirms the transitional and proliferative nature of the cells in cluster 3. The pathway analysis thus serves as a validating cross-reference, substantiating the functional insights derived from the UMAP analysis.

Using *PCLAF* as a marker for Cluster 3 we determined the temporal dynamics to understand how the cluster change over different stages of the menstrual cycle. Cluster 3 appeared at the mid-proliferative, peaked at the late secretory and decline rapidly afterwards (Fig. 4F). Very few cells from this cluster are observed in decidual samples (Fig. 4F). Based on these findings, we define cluster 3 as Transitional uDCs (T-uDCs).

### 5. Identification of conventional tissue resident uDCs (C-uDCs) in Cluster 2

**Figure 5:**
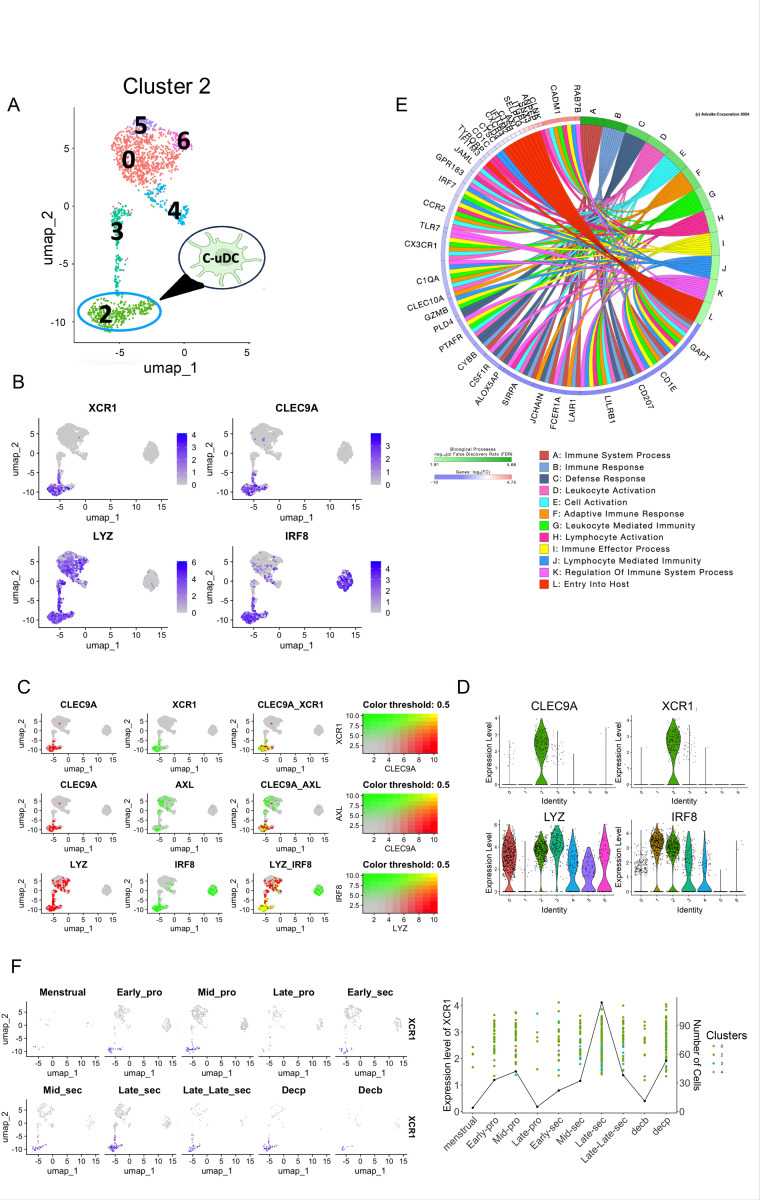
Identification and characterization of Conventional uDCs (C-uDCs) on the UMAP. **(A)** UMAP visualization uDCs with cluster 2 being identified as conventional uDCs. **(B)** UMAP projection displays the distribution of all analyzed cells (depicted in grey) alongside the subpopulation expressing conventional DC markers *CLEC9A, XCR1, LYZ* and *IRF8* (highlighted in blue). **(C)** Illustrates the subset of cells that co-express *CLEC9A* and *XCR1*, indicative of conventional DC identity (Top panel). Middle panel depicts a distinct group of cells characterized by the co-expression of both progenitor (*AXL*) and conventional DC markers (*CLEC9A*), representing a potential DC subset arising from the resident progenitor DCs. Bottom panel shows co-expression of *LYZ* and *IRF8* in the cluster 2, both the markers used for isolation of uDCs from all immune cells. **(D)** Violin plots quantifying the expression level and cluster specificity for the conventional uDC marker genes. **(E)** Circos plots showing the Biological processes for the C-uDC cluster along with the genes associated with each pathway. **(F)** UMAP showing the expression of the marker gene *XCR1* across 10 phases of the menstrual cycle, from the Menstrual stage through to Decidualization (Decp and Decb), indicating dynamic changes in gene expression. Alongside is shown a line graph combined with a scatter plot overlay depicting the expression level of *XCR1* (1^st^ y-axis) and number of cells expressing the gene (2^nd^ y-axis) across different stages of the menstrual cycle (x-axis). Each point represents the expression level of a gene in a particular cluster, while the line graph emphasizes the overall expression trend, and the expression peaks in late-secretory (WOI) period.

Having identified the progenitor and transitional uDC, we investigated whether these two clusters could generate conventional uDC. Cluster 2 was identified as a candidate for conventional uDCs based on shared expression of *LYZ* with P-uDC and T-uDC, and the shared expression of *IRF8* with PB-uDC (Fig. 5B). This suggests that these two clusters 0 and 3 (P-uDCs and T-uDCs) and cluster 2 have a tissue resident origin, while PB-uDCs and cluster 2 share some maturation characteristics.

GO analysis of differentially expressed genes that were significant (P < 0.05), and had a log fold change of at least 0.6, revealed some shared biological pathways between cluster 2 and PB-uDCs, indicating functional similarities (Fig. 5E and Fig. 2D). Specific markers for cluster 2, identified from the Bubble Plot (Supp. Figure 1), included *CLEC9A* and *XCR1*, distinguishing it from PB-uDCs (Supp. Figure 1 and Fig. 5B). The co-expression of *CLEC9A* and *XCR1* further confirmed that cluster 2 represents conventional uDCs (Fig. 5C).

Violin plots quantified the gene expression across different clusters, highlighting their significant expression in cluster 2 (Fig. 5D). These findings support the identification of cluster 2 as conventional uDCs (C-uDCs). Temporal analysis of cluster 2 during the menstrual cycle revealed that C-uDCs were detected during early-mid proliferative, peaking in the late secretory stage, which coincides with the Window of Implantation (Fig. 5F), suggesting a potential role during embryo implantation.

### 6. Identification of tissue resident uDCs with migratory potential

**Figure 6:**
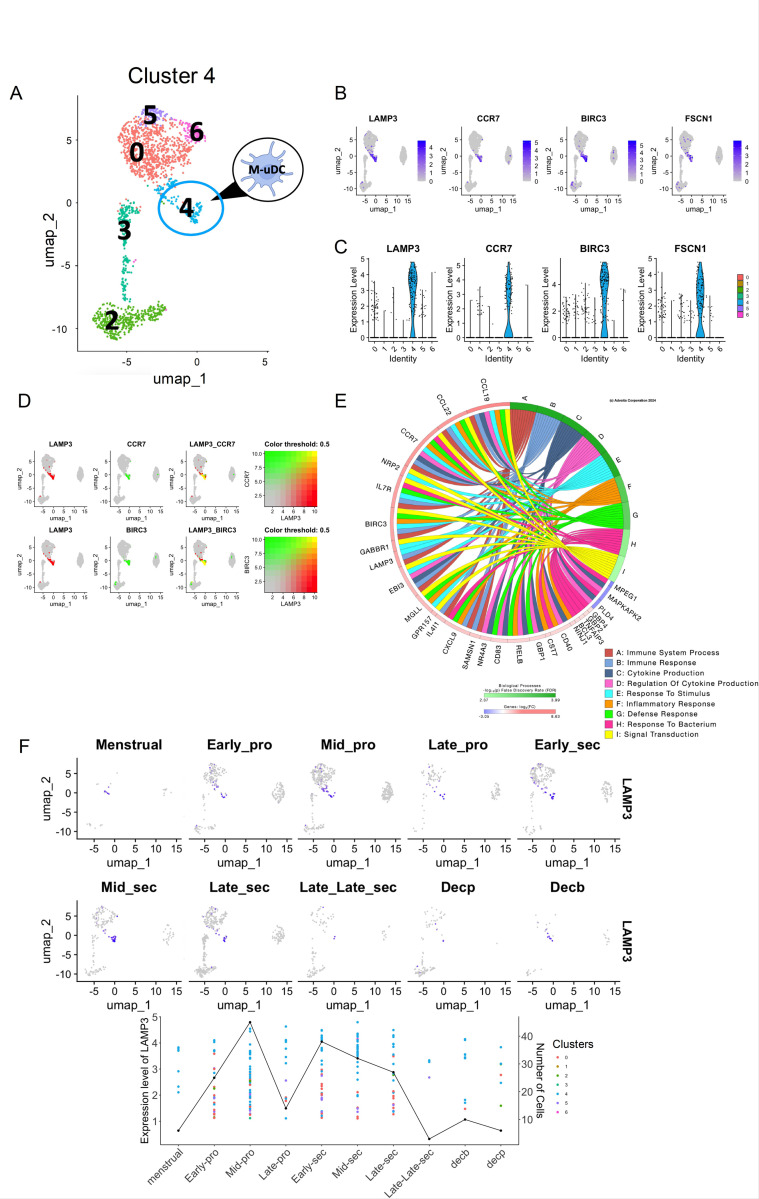
Identification and Characterization of Migratory uDCs in the human endometrium. **(A)** UMAP visualization of uDCs, with cluster 4 identified as migratory uDCs (M-uDCs). **(B)** UMAP displaying the distribution of all analyzed cells (grey) and the sub populations expressing the migratory DC markers *LAMP3, CCR7, BIRC3* and *FSCN1* (highlighted in blue). **(C)** Violin plots quantifying the expression levels and cluster specificity for the migratory uDCs marker genes *LAMP3, CCR7, BIRC3* and *FSCN1*. **(D)** Illustrates the subset of cells co-expressing *LAMP3* with *CCR7* and *BIRC3,* indicative of migratory identity. Co-expression analysis is shown with color thresholds to highlight the specific gene interactions. **(E)** Circos plots showing the biological processes for the M-uDC cluster along with the genes associated with each pathway. **(F)** UMAP showing the expression of the marker gene *LAMP3* across 10 phases of the menstrual cycle, from the menstrual stage through to decidualization (Decp and Decb). Below is a line graph combined with scatter plot overlay depicting the expression levels of *LAMP3* (1^st^ y-axis) and the number of cells expressing the gene (2^nd^ y-axis) across different stages of the menstrual cycle (x-axis). Each point represents the expression level of *LAMP3* in a particular cluster, while the line graph emphasizes the overall expression trend, with expression peaks during the mid-proliferative to late secretory phases with some presence in early decidual tissues.

A major function of DCs is antigen presentation to T-cells in the lymph node to induce either tolerance or T cell activation (35). We hypothesized that one of the identifed clusters within the tissue residents uDCs could reveal a transcriptome signature indicative of migratory/antigen presentation potential. To test our hypothesis, we focused on cluster 4 (Fig. 6A) and examined the marker genes. We identified *LAMP3, CCR7, BIRC3* and *FSCN1* specifically expressed in cluster 4 (Fig. 6B and Supp Figure 1). *LAMP3* is involved in the lysosomal function and membrane trafficking and is a marker for mature DCs, particularly those that migrate to lymph nodes (36). *CCR7* is crucial for the migration of DCs to lymph nodes, guiding them from peripheral tissues to the lymphoid organs (36) (10). *BIRC3*, also known as *cIAP2*, influences the survival of DCs, ensuring that they live long enough to effectively migrate and present antigens (36, 37). In migratory DCs, *FSCN1* facilitates the formation of DC projections (dendrites), aiding in their motility and interaction with T cells in the lymph nodes (38). The specific expression of these markers in cluster 4 was quantified using Violin Plots (Fig. 6C), and their co-expression is shown (Fig. 6D).

To understand the biological function of this cluster, we extracted all significant DEGs (P < 0.05, and log fold change of at least 0.6) and conducted a pathway analysis using iPathway. The main pathways expressed in cluster 4 are related to immune system process, immune response, cytokine production, regulation of cytokine production and signal transduction (Fig. 6E). The top genes differentially expressed in this cluster include chemokines (*CCL19, CCL22, CXCL9*), cytokines (*IL4l1*), and chemokine receptors (*IL7R*). Transcriptional factors such as *RELB*, are highly expressed in this cluster and are known to be essential for the regulation of T reg differentiation (39). In summary, this gene signature further suggests a mature, antigen processing uDC phenotype. Temporal analysis of cluster 4 revealed these cells are present in the mid proliferative phase and decrease at the late-late secretory phase. Interestingly, these cells are detected in the decidua of early pregnancies (Fig. 6F). Accordingly, we define cluster 4 as Migratory uDCs (M-uDCs).

### 7. Presence of potential Antigen presenting resident uDC (A-uDCs)

Clusters 5 and 6 follow a different path of differentiation compared to clusters 3 and 4 (Fig. 3E), suggesting shared biological functions. To identify specific markers for cluster 5, we used the Bubble plot (Supp Fig. 1) and identified *IL22RA2, CDH17, CD207* and *SYT2* genes as potential markers. *IL22RA2, SYT2* and *CDH17* are found expressed only in cluster 5; while *CD207* is shared with clusters 0 and 6; indicating *CD207* as a marker for this line of differentiation (Supp. Figure 2). The presence of *IL22RA2, CDH17, CD207*, and *SYT2* in cluster 5 suggests an antigen-presenting function, as these genes are crucial for antigen processing and presentation. *IL22RA2* encodes a receptor for interleukin 22 (*IL-22*), involved in inflammatory responses and tissue repair, influencing DCs maturation and function (40). *CDH17* is crucial for DCs migration to lymph nodes and interaction with T cells (41, 42).

UMAP analysis showed specific expression of these genes in cluster 5 (Supp Figure 2B) and corroborated with quantified expression levels using violin plots (Supp. Figure 2C). Co-expression showed *CDH17 + CD207*, and *CDH17 + IL22RA2* specificity to cluster 5 (Supp. Figure 2D). iPathway analysis (for significant DEGs (P < 0.05), and log fold change of at least 0.6) revealed “response to stimulus” as significant (Supp. Figure 2E). Temporal analysis showed increased cell numbers in early proliferative, peaking in early-secretory phases, and declining through the late-sec phases, and non-existent in the decb stage (Supp. Figure 2F). These characteristics define cluster 5 as Antigen-presenting uterine DCs (A-uDC).

Cluster 6 shares several markers with cluster 5, including *HLA-DBQ2*, involved in recognizing and presenting common self-antigens to induce tolerance (Supp. Figure 3B-C); further supporting the premise that these clusters may play a role on tolerance to paternal antigens during conception. The temporal distribution of these cells through the menstrual cycle has similar pattern as cluster 5 (Supp. Figure 3F).

*NONO* (Non-POU Domain-Containing Octamer-Binding Protein) and *EIF5A* are expressed in all uDC clusters (Supp. Figure 3C). *NONO* is involved in the regulating immune-related gene expression and synthesis of pro-inflammatory cytokines (43). *EIF5A* plays a critical role in DCs maturation. Thus, *NONO* and *EIF5A*, along with *LYZ* and *IRF8*, can be used as general markers for uDC.

### 8. Dynamic sub-cellular heterogeneity of DC in the human endometrium

**Figure 7:**
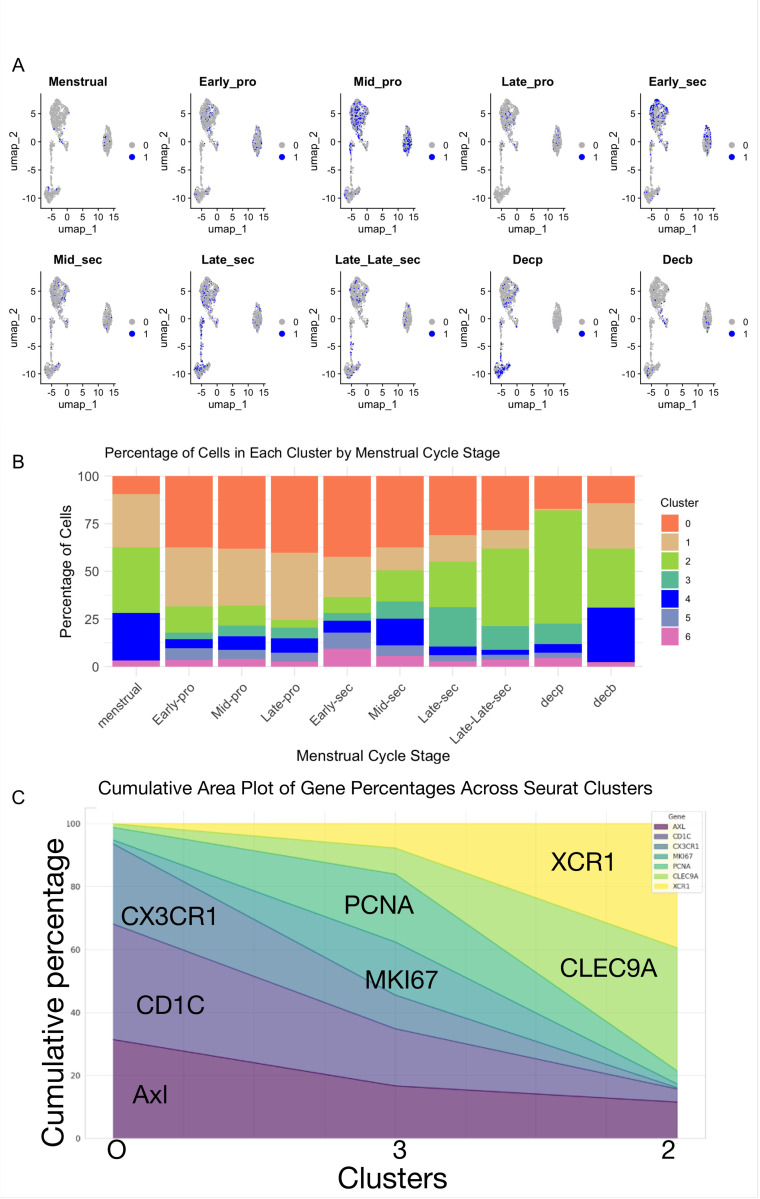
Sequential transition from progenitor to differentiated states in uDCs maturation. **(A)** UMAP visualization of cells from each stage of the menstrual cycle. Grey represents all cells, while blue highlights the cells coming from the specific stage indicated on the top of the UMAP. **(B)** Histogram showing the distribution of cells from different menstrual cycle stages across each cluster of the UMAP. The y-axis represents the percentage of cells, and x-axis indicates the menstrual cycle stage. The different colors in the histogram bar represent different clusters which are indicated on the right. **(C)** Area plot showing cumulative percentage of cells (y-axis) expressing key genes for progenitor, transitional and conventional uDCs shown on x-axis. AXL has a marked presence in each stage. In proliferative stages, uDCs express AXL along with CD1C and CX3CR1 genes, which are lost by the cells in later stages of differentiation. The cells gain proliferative markers such as PCNA and MKI67 in their differentiating stages and then lose these proliferative markers to gain the CLEC9A and XCR1 conventional uDCs markers.

The cyclic hormonal nature of the human endometrium influences the nature and number of immune cells present within the female reproductive tract (44). To determine the impact of hormone-regulated changes on the global presence of DCs and their specific clusters, we employed UMAP analysis for detailed visualization. This analysis revealed a broad distribution of cells in each menstrual stage; with varying numbers and types throughout the menstrual cycle (Fig. 7A). During menstruation, very few cells were localized in clusters 0, 1, 2, and 4. In the proliferative phase, there is an early increase in cluster 0 (P-uDC) and the recruitment of peripheral DCs (cluster 1, PB-DC). The number of PB-DCs is high in the mid-proliferative phase and decreases afterward, becoming minimal in the rest of the cycle (Fig. 7). Clusters 3 (T-uDCs) and 2 (C-uDCs) are present mainly during the secretory phase, peaking in the late secretory phase. If pregnancy occurs (Decp and Decb), cluster 2 (C-uDCs) will remain present in the decidua; however, in the absence of pregnancy, their number will decrease and disappear during menstruation. Clusters 5 and 6 are mainly present during the early secretory phase. Cluster 4 is detected in the mid-proliferative phase and remains present until the late secretory phase (Fig. 7A).

The percentage of cells in each cluster varied in relation to the days of the menstrual cycle. Cluster 0 is present throughout the menstrual cycle, with its percentage increasing during the proliferative and early secretory phases. Cells differentiated from cluster 0 appear in the proliferative phase and increase during the secretory and early pregnancy phases. The percentage of cells in cluster 1 increase during the proliferative phase, decreasing afterwards. The percentage of cells from cluster 4, 5 and 6 increase only during mid-secretory and are present during early pregnancy in the decidua basalis but not in parietalis (Fig. 7B).

This finding suggests that sub-cellular heterogeneity of DCs within the endometrium remains remarkably dynamic throughout the menstrual cycle, challenging previous assumptions about presence of a single type of DC in the human endometrium.

### 9. Developmental trajectory from progenitor to differentiated states in uDCs

Our next objective was to map the developmental trajectory of tissue resident uDCs. Using the Slingshot algorithm, we identified a developmental trajectory of uDCs starting from cluster 0, progressing through cluster 3, and culminating in cluster 2 (Fig. 3E). We use *AXL +* as markers of cluster differentiation since it was expressed in all 3 clusters (0, 3 and 2). High percentages of *AXL+/CX3CR1+/CD1A +* are found in cluster 0, and their percentage decreases as they progress through cluster 3 and 2 (Fig. 7C). These progenitor cells transition to a proliferative state in cluster 3, indicated by high percentages of *AXL+/MKI76+/PCNA+*, decreasing as they progress into cluster 2. As the percentage of cells expressing *MKI67+/PCNA +* decrease, we observe an increase in the percentage of *CLEC9A+/XCR1 +* cells found in cluster 2 (Fig. 7C). This trajectory highlights a well-defined pathway of tissue resident uDCs maturation that is consistent across the menstrual cycle, reinforcing the notion of a complex yet orderly dendritic cell developmental process.

### 10. Inflammatory response for implantation is distinct from other inflammatory responses

Inflammation plays a crucial role in uterine homeostasis, but there are different types of inflammatory signatures. We aimed to identify the specific inflammatory responses each cluster contributes to the endometrial milieu. Detailed investigation into the genes driving these functions (45, 46) revealed that clusters 0, 1, 2 and 4 exhibit inflammatory response hallmark functions. We extracted genes associated with inflammatory response pathways from these clusters and generated a heatmap to visualize the gene expression patterns (Supp. Figure 4). Our heatmap analysis revealed distinct and unique inflammatory signatures for each cluster. We then analyzed the functions of most-highly upregulated genes in each cluster compared to other clusters (indicated by arrows) (Supp. Table 1). The inflammatory genes uniquely expressed in cluster 0 are primarily associated with immune regulation of the adaptive and innate immune responses.

Cluster 2 was most prominent during the late-secretory phase, also known as the Window of Implantation, as observed from the UMAP. Analyzing the specific genes identified in cluster 2’s inflammatory signature, we found pro-inflammatory mediators uniquely expressed, including *XCR1, SOD1, BCL6* and *HDAC9*. These genes are associated with the process of implantation and their regulation, such as *BCL6*, is indicative of pregnancy complications like miscarriage. Based on these findings, we propose that cluster 2 plays a pivotal role in mediating implantation and early pregnancy (Supp. Figure 4 and Supp. Table 1).

### 11. uDCs Inter-cluster immune interaction

**Figure 8:**
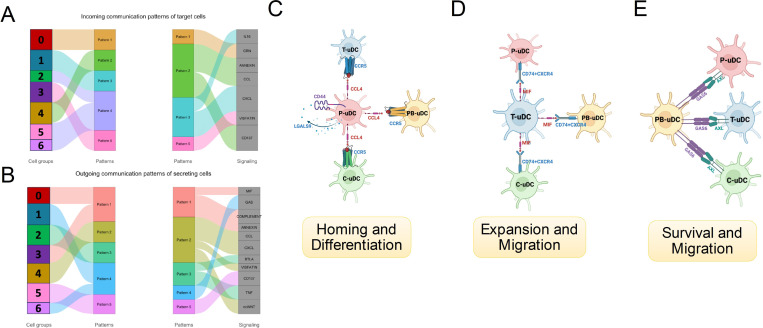
Receptor-Ligand interaction between different sub-types of the uDCs. **(A)** Visualization of intercellular communication within the uDC network, illustrated through river plots that delineate both incoming and outgoing signaling patterns. Figure highlights the incoming communication pathways to target cells, mapping how various signals are received by different uDC subtypes from their counterparts. **(B)** Complements this by showing the outgoing communication from these cells, detailing the types of signals transmitted outward and their respective targets across the cellular network. **(C and D)** graphically represent the expansion, migration, homing, and differentiation stages of uDCs, showcasing specific receptor-ligand pairs such as *CD74/CXCR4* and *CCL4/CCR5*that facilitate these processes. The diagrams provide a concise view of how distinct signaling mechanisms contribute to the spatial and functional organization of uDCs. **(E)** details the interactions within the PB-uDC cluster, emphasizing the survival and migration pathways mediated by key molecules like *GAS6* and *AXL*.

Our next objective was to analyze the communication/signaling between various sub-types of the uDCs by dissecting the ligand-receptor signaling networks between the various subtypes of uDCs (CellChat, Suppl. Figure 5) (47). The signaling received (incoming) by the 7 sub-types of uDCs were classified in mainly five patterns (Fig. 8A) and include *IL16, GRN, ANNEXIN, CCL, CXCL, VISFATIN*, and *CD137* (Fig. 8A). The signaling sent (outgoing) from the 7 sub-types of uDCs were also classified in five major patterns and include *MIF, GAS, COMPLEMENT, ANNEXIN, CCL, CXCL, BTLA, VISFATIN, CD137, TNF*, and *ncWNT* (Fig. 8B).

First, we analyzed the communication pattern of *LGALS9-CD44*, which is essential for the homing of hematopoietic stem cells to their bone marrow niches, and was found uniquely on the P-uDCs; suggesting of autocrine signaling. The second pattern is represented by *CCL4/CCR4*; which identify only one cluster expressing *CCL4* (P-uDCs) and 3 clusters of uDCs expressing *CCR5*, T-uDCs, PB-uDCs and C-uDCs. This pathway is essential for the differentiation of DCs as well as in the recruitment of immune cells to sites of infection or inflammation and revels a communication between P-uDCs and T-uDCs, PB-uDCs and C-uDCs (Fig. 8C). The third pattern is represented by *MIF/CD74-CXCR4* and links T-uDC (in cluster 3) expressing *MIF*, with C-uDCs, PB-uDCs and P-uDCs expressing the MIF receptors *CD74-CXCR4*. *MIF/ CD74-CXCR4* interaction is associated with cell migration and promotes antigen presentation (Fig. 8D). The fourth pattern involves *GAS6-AXL* pathway linking the PB-uDCs, expressing *GAS6* and all the tissue resident uDCs subtypes expressing *AXL* (48, 49). This pattern demonstrates a direct and unique communication between the resident and recruited DCs (Fig. 8E).

### 12. CITE-seq validates the presence of major subsets of uDCs

**Figure 9:**
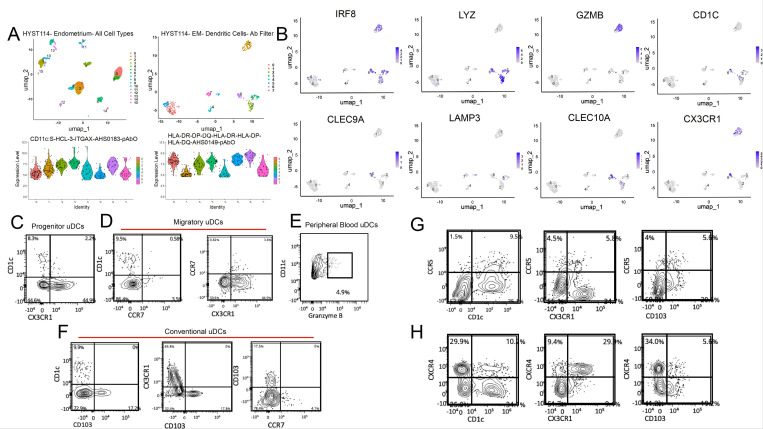
Characterization and Distribution of Uterine Dendritic Cells (uDCs) in Human Endometrium. **(A)** UMAP plots and Violin Plots: Left panel: UMAP visualization of all cell types in the endometrium. Each color represents a different cell cluster identified by unsupervised clustering. Right panel: UMAP visualization specifically of DCs within the endometrium after applying an antibody filter. Clusters are colored to indicate different DC subpopulations. Bottom panel: Violin plots showing the expression levels of specific markers (CD11c, SLC15A3, ITGAX, ASH1B, A3B) and major histocompatibility complex (MHC) molecules (HLA-DR, HLA-DQ, HLA-DO, HLA-DP) across different identified clusters. **(B)** UMAP feature plots depicting the expression of specific genes: IRF8, LYZ, GZMB, CD1C, CLEC9A, LAMP3, CLEC10A and CX3CR1.**Flow Cytometry Plots in (C)** shows the expression of CD1c and CX3CR1 on progenitor uDCs. **(D)** Migratory uDCs displaying the expression of CD1c, CCR7, and CX3CR1 identifying their surface marker profile. **(E)** illustrates the expression of CD11c and Granzyme B in peripheral blood uDCs, indicating the proportion of cells with cytotoxic potential. **(F)** shows expression profiles of CD1c, CD103, CX3CR1, and CCR7 in conventional uDCs. **(G)**depicts the expression of CCR5 in combination with CD1c, CX3CR1, and CD103 on uDCs. **(H)** shows the expression of CXCR4 in combination with CD1c, CX3CR1, and CD103, indicating the presence and distribution of this chemokine receptor on uDCs.

To validate our RNAseq data, we determined protein expression of markers definitory of DCs and individual subsets. First, we reanalyzed our CITE-seq performed on endometrial single-cell suspensions to link surface protein expression with key genes identified by our RNAseq analysis (50). Using antibody sequencing information, we identified the DC population by selecting CD45+, lineage negative cells that expressed CD11c and HLA-DR. These cells formed multiple clusters all of which displayed surface expression of canonical markers for DCs (CD11c^high^, HLA-DR^high^, CD83) and tissue residency (CD69) (Fig. 9A). Analysis of specific genes revealed IRF8 and LYZ expression (Fig. 9B), consistent with our RNAseq data. We identified PB-uDCs (IRF8, GZB), T and P-uDCs (CX3CR1, CD1C, CLEC10A), C-uDCs (CLEC9A) and a small number of migratory-uDCs (LAMP3).

Next, we used spectral flow cytometry to further validate individual DC subsets. Gating on CD45+, Lin-, CD11c^high^, HLA-DR^high^ (Supp. Figure 6), we identified a large proportion of DCs that expressed either CD1c or CX3CR1, and a small subset of DCs that co-expressed both markers, consistent with the presence of progenitor and transitional DCs (Fig. 9C). Migratory DCs were identified by CCR7 expression, and were different from CD1c + DCs, but we detected patient-dependent co-expression with CX3CR1 (Fig. 9D), supporting that migratory DCs originate from P-uDCs. Conventional DCs were identified by CD103 expression (51), and these DCs did not co-expressed CD1c, CX3CR1, or CCR7, confirming they are a separate DC subset (Fig. 9F). CD1a + DCs were also identified and a subset of them co-expressed CD1c, likely corresponding to the antigen presenting cell cluster identified with RNAseq. Finally, we performed intracellular staining and identified GZMB + DCs (Fig. 9E), indicating the presence of PB-uDCs.

To validate our DC interactions data, we next determined expression of CCR5 and CXCR4. As seen in Fig. 9G–9H, P, T and C-DCs (CD1c+, CX3CR1+, CD103+) express CCR5 and CXCR4, allowing interactions with CCL4 and CD74 respectively, as predicted by our CellChat data.

### 13. Multiplex Immunohistochemistry validates the presence of major sub-types of uDCs

**Figure 10:**
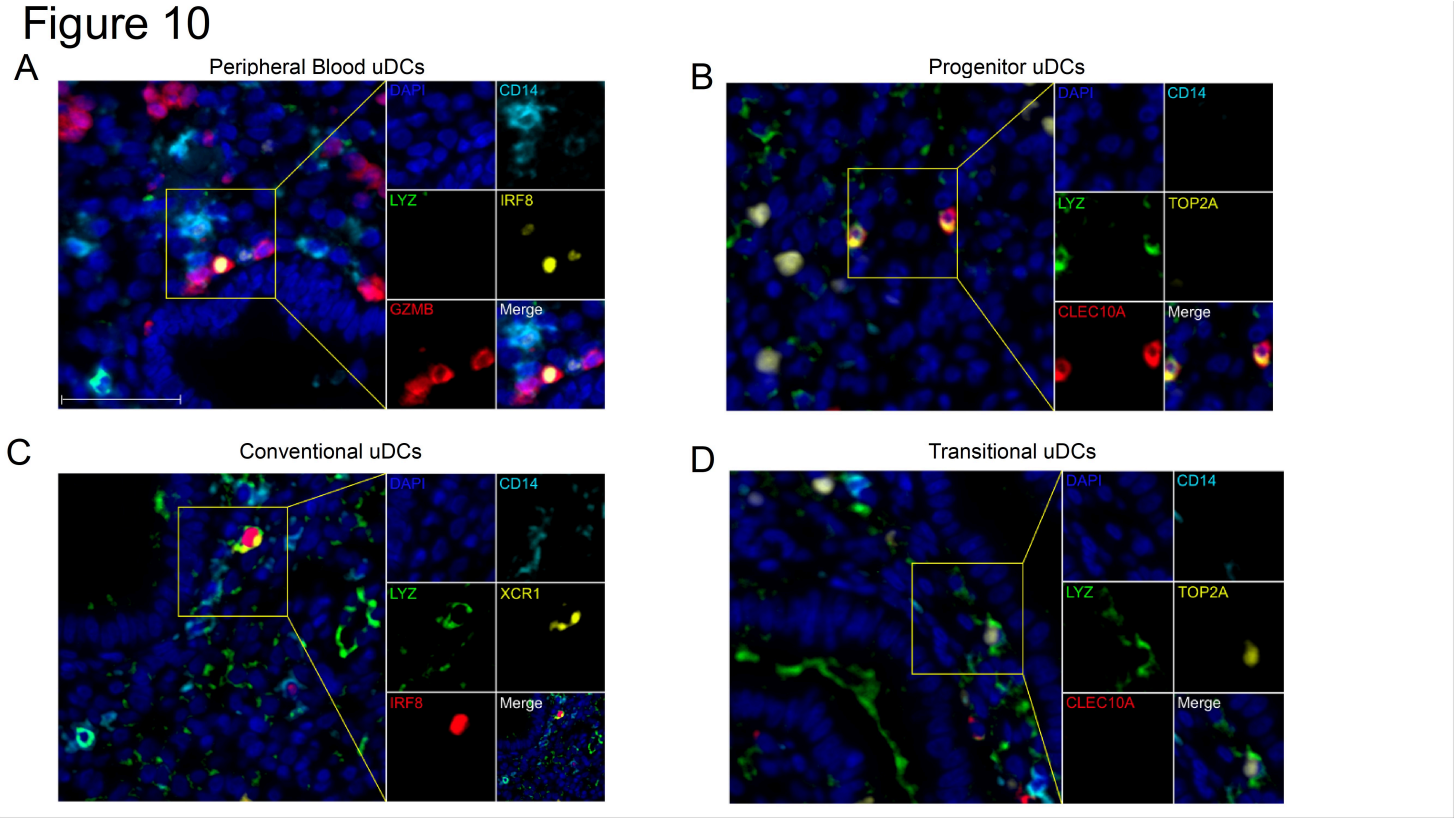
Validation of Uterine Dendritic Cell (uDC) Sub-Types in the Mid-Secretory Phase of the Endometrium by Multiplex Immunohistochemistry (IHC). **(A)** Peripheral Blood uDCs: IHC staining for various markers such as DAPI (blue) for nuclei, *CD14* (cyan), *GZMB* (red), *IRF8* (yellow), and *LYZ* (green) is shown. Cells stained for *IRF8* and *GZMB*, lacking CD14 and *LYZ*, indicating peripheral blood-derived uDCs. **(B)** Progenitor uDCs: Cells stained for various markers DAPI (blue) for nuclei, *CD14*(cyan), *CLEC10A* (red), *TOP2A* (yellow), and *LYZ* (green) are shown. Cells stained for *CLEC10A* and *LYZ*, lacking *CD14* and *TOP2A,* indicating progenitor uDCs. **(C)** Conventional uDCs: IHC staining for marker genes such as DAPI (blue) for nuclei, *CD14* (cyan), *XCR1*(yellow), *IRF8* (red), and *LYZ* (green) are shown. Cells stained for *XCR1, IRF8*, and *LYZ*, lacking *CD14,* indicating conventional uDCs. **(D)** Transitional uDCs: IHC staining for marker genes such as DAPI (blue) for nuclei, *CD14* (cyan), *CLEC10A* (red),*TOP2A* (yellow), and *LYZ* (green) are shown. Cells stained for*TOP2A* and *LYZ*, lacking *CD14* and *CLEC10A,* indicating transitional uDCs. Scale bar, 50 μm.

Multiplex immunohistochemistry (IHC) was performed to validate the presence of four major sub-types of uDCs, (progenitor uDCs, peripheral blood-derived uDCs, transitional uDCs, and conventional uDCs), in endometrial sections from the mid-secretory phase. We designed a panel of markers to test for each sub-type based on the identified genes. For peripheral blood derived uDCs we used *IRF8* and *GZMB*; for Progenitor uDCs *CLEC10A*; for Conventional uDCs *XCR1*, and for Transitional uDCs *TOP2A. LYZ* and *IRF8* were used as general markers for DC, *CD14* for macrophages and *DAPI* for nuclear staining. As shown in Fig. 10A, we identify a *CD14*^*−*^*/IRF8*^*+*^*/GZMB*^*+*^*/LYZ*^*−*^ cells corresponding to peripheral blood recruited DCs. Similarly, we detected cells characterized as *CD14*^*−*^*/CLEC10A*^*+*^*/LYZ*^*+*^*/TOP2A*^*−*^ corresponding to progenitors uDCs (Fig. 10B), conventional DCs as *CD14*^*−*^*/LYZ*^*+*^*/IRF8*^*+*^*/XCR1*^+^ (Fig. 10C) and Transitional uDCs as *CD14*^*−*^*/LYZ*^*+*^*/CLEC10A*^*−*^*/TOP2A*^*+*^ (Fig. 10D).

These findings confirm the presence of distinct sub-types of uDCs present in the mid-secretory phase of the endometrium, corroborating the insights from scRNAseq analysis.

## Discussion

This study provides the first in depth characterization of bona fide DCs in the human endometrium and reveals the existence of distinct subsets with differential functions that are tightly regulated by the menstrual cycle. Employing single-cell RNA sequencing on uterine tissues collected across different menstrual phases and during early pregnancy, and validated with CITE-seq, we show that, in addition to DCs recruited from peripheral blood, the majority of uDCs differentiate from resident progenitor uDCs. The presence of resident progenitor uDCs suggest a self-renewing source essential for the preparation of the endometrium for embryo implantation and successful pregnancy.

The human endometrium undergoes complete tissue remodeling every menstrual cycle, involving multiple cell types, including epithelial, stroma and immune cells (5, 52–54). The regenerative capacity of the endometrium is attributed to stem/progenitor cells, which resides in both the epithelium and stromal cell components of the basalis layer (55, 56). Traditionally, the origin of immune cells has been attributed to active recruitment from the peripheral blood. However, this concept has been challenged with recent data which suggests the existence of immune progenitor cells within the human endometrium (57). The findings described in this study show that, although there is a recruited component of the uDC population, there is also a unique and function specific tissue residents uDCs.

uDCs represent a small percentage of the endometrial cellular component but play multiple roles including tissue renewal, antigen presentation, and preparation of the endometrium for successful embryo implantation and placentation (46, 58, 59). They contribute to fetal-maternal tolerance by regulating T cell activation, paternal antigen presentation, maintaining fetal-maternal tolerance and creating a unique inflammatory environment for embryo implantation (60, 61). However, it was unclear how these multiple and diverse functions are performed by the two classical classifications of DCs: conventional DCs (cDCs) and plasmacytoid DCs (pDCs). Therefore, we tested the hypothesis that within the uDC population, sub-clusters with specific functions must exist.

Using single-cell transcriptomic analysis of the *LYZ+/IRF8 +* DCs, we identified seven uDC subtypes, including cells with characteristics of resident progenitor cells and peripheral blood-derived DCs. We chose these two transcription factors because *IRF8* is expressed by DCs that originate from hematopoietic progenitors in the bone marrow, and *LYZ* is a marker for circulating progenitors of conventional DCs (22, 62). Interestingly, we observed that all resident uDCs exhibit *AXL* expression. Notably, cluster 1, which we identified as being recruited from peripheral blood, lacks this characteristic. Therefore, we propose that *AXL* expression could serve as a potential marker for distinguishing endometrial resident DCs from those derived from peripheral blood.

Cluster 1, identified as PB-uDC, exhibits a distinct gene expression profile similar to that of peripheral blood plasmacytoid DCs. This includes high *IRF8* expression and involvement in specific functional pathways such as allograft rejection, TNF-α signaling, cell motility, movement, and locomotion. These findings provide additional evidence that these cells are actively migrating and being recruited. Furthermore, a 44% similarity with the peripheral blood cluster suggests that while these cells originate from the peripheral blood, they undergo modifications in the tissue environment, leading to differences in their gene expression profiles.

Having identified clusters of tissue residents uDCs, we then sought to distinguish progenitors and differentiated uDCs. Using *AXL* as the marker for tissue residents uDCs we pinpointed cluster 0, as the tissue progenitor uDCs (P-uDCs). This cluster is characterized by the expression of markers such as *KLF4, CX3CR1, CLEC10A*, and *CD1C* and exhibit a trajectory of differentiation, transitioning through cluster 3 (transitional/proliferative uDCs, T-uDCs) and culminating in either cluster 2 (conventional uDCs, C-uDCs) or a second trajectory to clusters 4, 5 and 6.

We identified Cluster 3 as a transitional type of uDC (T-uDCs) showing high expression of *PCLAF, TOP2A, MKI67, TYMS*, and *TK1*, genes associated with a proliferative state. These cells appear during the proliferative phase of the menstrual cycle, suggesting a potential role on the regeneration of the endometrium following menstruation.

Cluster 2 revealed characteristics of conventional uDCs, (C-uDCs) marked by *CLEC9A* and *XCR1* expression. This cluster aligns with conventional DC functions, particularly antigen presentation and the expression of a unique set of inflammatory mediators, necessary for the preparation of the endometrium for embryo implantation. Indeed, in animal studies and in human samples we demonstrated a specific inflammatory signature necessary for the preparation of the epithelium of the lumen to become receptive to the embryo(20, 63). This inflammatory signature, responsible for the removal of the mucin-16 layer, expression of adhesion molecules, osteopontin (*SPP1*) and *CD44*, and increase in vascularity, was mainly determined by DCs (20, 63). In this study, we identified C-uDCs as the specific uDC cluster responsible for establishing this unique inflammatory signature required for the success of embryo implantation. Furthermore, the number of C-uDCs increase during the late secretory phase, coinciding with the window of implantation.

Clusters 4, 5 and 6 were identified as immune modulatory uDCs. Cluster 4 showed characteristics of migratory uDCs (M-uDCs), depicted by the expression of *LAMP3, CCR7, BIRC3*, and *FSCN1*, and underscores the dynamic nature of M-uDCs in immune surveillance and response to foreign antigens within the endometrium. Chemokine receptor such as *CCR7*, enables the migration of uDCs to the lymph nodes (10). These cells are prominent during the mid-proliferative to late secretory phases and are detected in early pregnancy decidual tissues, indicating their potential role in maternal-fetal tolerance. Clusters 5 and 6, which share several markers with cluster 4, appear to be involved in antigen presentation and immune regulation. They are characterized by specific markers such as *IL22RA2, CDH17, CD207*, and *SYT2.*

Early studies, such as those by Rodriguez-Garcia’s group(58, 64), suggested the presence of distinct subsets of DCs based on the detection, by flow cytometry, of CD11c, CD11b, CD14, CD1c, and CD103 in the female reproductive tract and their role in the protection against viral infections, such as HIV(50, 59). None of those studies looked at the different phases of the menstrual cycle.

An important finding of this study is that the presence of the different uDC clusters varies according to the changes of the human endometrium during the different phases of the menstrual cycle, which is in line with previous studies describing changes in the uDC population according to the phase of the menstrual cycle and the layer of the endometrium (65) (66). Previous studies were limited to the use of few markers, such as CD1a and CD83 for immunocytochemistry or flow cytometry; therefore, constraining the possibility to identify all the different sub-populations identified in this study.

We observe that PB-uDCs (cluster 1), are recruited from peripheral blood during the proliferative phase, which aligns with previous studies showing increased numbers of recruited DCs during the proliferative phase, indicative of a hormonal influence on the recruitment of peripheral uDCs (50, 65–67). In contrast, the expansion of tissue resident uDCs occurs during the secretory phase which may be hormone independent and might be associated with the preparation of the endometrium for embryo implantation and placentation.

Previously, we and others demonstrated that endometrial biopsies lead to an increase in pro-inflammatory cytokine levels, which was associated with successful pregnancy outcomes (20, 68). This outcome was related to the activation of DCs (63). Indeed, depletion of dendritic cells (DCs) in mice, prior to implantation, is linked to implantation failure (17, 69). Additional studies in different species have further strengthened the concept that a specific inflammatory signature is necessary for the induction of molecular and functional changes of endometrial cells, including epithelial and stromal during the process of embryo implantation (70, 71). By extracting and analyzing genes associated with inflammatory pathways, we identified that clusters 0, 1, 2, and 4 exhibit inflammatory responses as hallmark functions. As indicated above, Cluster 2 stood out as particularly prominent during the late-secretory phase, coinciding with the window of implantation. The specific inflammatory mediators uniquely expressed in this cluster, including *XCR1* (72), *SOD1* (73), *BCL6* (74), and *HDAC9* (75), critical for the implantation process. Furthermore, this provides insights into better understanding the pathophysiology and the cellular origin associated with pregnancy complications such as implantation failure and miscarriage characterized by dysregulation of some of these gens, such as *BCL6*. *BCL6* is crucial for the survival and differentiation of stem/progenitor cells within the endometrium and also plays a role in the differentiation, migration, and invasion of trophoblastic cells. (72–75).

PB-uDCs (clusters 1) and M-uDCs (cluster 4) also contribute to the inflammatory landscape, each with distinct gene expression profiles. PB-uDCs’ inflammatory genes are involved in pathways that likely support the recruitment and activation of peripheral immune cells, whereas M-uDCs’ inflammatory signature suggests a role in antigen presentation and migration, essential for effective immune surveillance and response. These findings collectively underscore the importance of diverse inflammatory responses among uDC subtypes in creating a balanced and dynamic endometrial environment, crucial for successful implantation and pregnancy maintenance.

The clinical implications of our findings are significant. By identifying specific uDC subsets that contribute to endometrial receptivity and implantation, we open new opportunities for therapeutic interventions aimed at enhancing uDC function in women with reproductive disorders. For example, targeting the AXL + progenitor uDCs or modulating the inflammatory environment created by conventional uDCs could offer new strategies for improving implantation success in assisted reproductive technologies (ART).

However, our study is not without limitations. While we identify a resident progenitor uDC population and map differentiation pathway, the findings are based on gene expression and trajectory analysis without direct functional validation. Experimental methods like lineage tracing are needed to confirm progenitor capacity. Additionally, our pathway and receptor-ligand interaction analyses suggest potential roles in reproductive health, yet these insights remain speculative without experimental evidence. The inflammatory signature linked to implantation also requires functional validation to confirm its relevance.

Functional studies on DCs, however, can only be conducted in mouse models, limiting the direct generalizability of these findings to humans. Despite this, we have demonstrated the relevance of DCs during implantation using animal models, providing valuable insights into their potential roles. Another limitation of our study is the variability introduced by collecting samples from multiple patients, as obtaining repeated samples from the same individuals would be neither ethical nor practical. Endometrial biopsy is an invasive and painful procedure, and performing it multiple times poses significant risks to patients, including severe pain and discomfort, increased health risks, potential long-term damage to the endometrial lining, and the risk of developing Asherman’s syndrome, a condition with lasting health implications.

It is noteworthy that all published studies on endometrial sampling across the menstrual cycle follow similar designs, and, to our knowledge, no study has achieved a sample size as large as ours. We hope that our omics-based insights into uDC functionality will inform future research focused on functional validation, further elucidating the roles of these cells in reproductive health and potential therapeutic applications.

In conclusion, our findings fill a critical gap in understanding the origin, molecular characteristics, and roles of uDCs in the human endometrium. Our results reveal a dynamic and heterogeneous uDC landscape in the human endometrium, with distinct subpopulations playing specialized roles in immune regulation, antigen presentation, and preparation for implantation. Using the single cells analysis in samples from the different phases of the menstrual cycle and validated by CITE-seq and immunocytochemistry we were able to provide a comprehensive mapping of the different uDC clusters with association of their potential function and time within the endometrium. These findings open new venues for further research into the therapeutic potential of targeting specific uDC subsets in reproductive health and disease.

## Material and Methods

### 1. Sample Collection and Ethical Considerations

The study was approved by the medical ethics committee of Shenzhen Zhongshan Obstetrics & Gynecology Hospital (formerly Shenzhen Zhongshan Urology Hospital), Shenzhen, China. All procedures and protocols involving human endometrium were approved by the institutional review boards (SZZSECHU-2020010). Written informed consent was obtained from all participants before the collection of endometrial biopsy specimens. The inclusion criteria for healthy women were ages 18–40, BMI 18–25 kg/m^2^, regular menstrual cycles (3–5 days every 25–35 days), natural menstrual cycles with no hormone stimulation, and negative serological tests for HIV, HBV, HCV, and syphilis. Exclusion criteria included a history of two or more abortions, three or more biochemical pregnancies, uterine pathology (endometritis, endometriosis, uterine polyp, adenomyosis, polycystic ovary syndrome, intrauterine adhesion), bacterial, fungal, or viral infection, and history of autoimmune or thyroid-related diseases.

### 2. Menstrual Cycle Staging

Endometrial tissues were classified according to the menstrual cycle stages as follows: days 1–3 (menstrual phase, sample size: 3), days 4–7 (early proliferative phase, sample size: 8), days 8–11 (mid proliferative phase, sample size: 8), days 12–14 (late proliferative phase, sample size: 5), days 15–18 (early secretory phase, sample size: 7), days 19–22 (mid secretory phase, sample size: 8), days 23–25 (late secretory phase, sample size: 13), days 26–28 (late-late secretory phase, sample size: 3), and early pregnancy (gestational weeks 5–8, sample size: 5).

### 3. Tissue Dissociation and Single-Cell Suspension

Endometrial tissues were digested in 2 mL RPMI-1640 medium containing 2 mg/mL collagenase IV (17104–019, Invitrogen) and 0.0125 mg/mL DNase I (D4527, Sigma) for 20 minutes at 37°C. The digested tissue was passed through a 70 μM cell strainer to obtain a single-cell suspension. Red blood cells were lysed using RBC lysis buffer for 10 minutes at room temperature, followed by washing with PBS.

### 4. Fluorescence-Activated Cell Sorting (FACS)

Single-cell suspensions were subjected to FACS using a BD FACSAria Fusion sorter. CD45 + cells (Biolegend, 304006) were sorted into 384-well plates (Eppendorf) for downstream single-cell RNA sequencing (scRNAseq).

### 5. Single-Cell RNA Sequencing (scRNAseq)

Sorted cells were processed for transcriptome library preparation following the MARS-seq2.0 protocol (76). Specifically, HISAT (version 0.1.6) with default parameters was used to map the reads to the human reference genome hg38, and reads with multiple mapping positions were excluded. Reads were associated with genes if they were mapped to an exon, using Homo sapiens Ensembl90 for reference. Reads were condensed into original molecules by counting the same unique molecular identifiers (UMI). The batches used exhibited low cross single-cell contamination (under 3%), confirmed through spurious UMI detection in empty wells.

### 6. Statistical Methodology and Power Consideration

While formal power analysis is challenging for scRNA-seq studies due to the unique nature of single-cell variability and sampling, our study was designed to achieve robust detection and characterization of rare cell types, specifically dendritic cells (DCs), which constitute less than 1% of the endometrial cell population. Our comprehensive single-cell sequencing approach, combined with deep sequencing depth and rigorous quality control, allowed us to reliably identify and analyze these rare DC populations.

To ensure statistical relevance, we employed the following methodology:

- Detection of Rare Cell Populations: The statistical power of our study was evidenced by the detection of rare uDC subpopulations within the CD45 + immune cell compartment. The combination of single-cell RNA-seq, CITE-seq, and stringent bioinformatics analysis allowed us to capture rare cell states effectively, demonstrating sufficient resolution to identify DCs reliably.
- Gene Filtering and Normalization: Genes expressed in fewer than three cells were filtered out to reduce noise. We performed log-normalization on the data and identified highly variable features to focus on biologically meaningful variation.
- Clustering and Differential Expression Analysis: Clustering was performed at a resolution of 0.5, which was empirically determined to capture distinct cell populations. Differential expression analysis used a minimum log fold change of 0.6 and an adjusted p-value threshold of 0.05, based on the Benjamini-Hochberg correction, to control for false discovery rates.

Through these methods, we ensured that our analysis was statistically robust and capable of capturing rare and functionally relevant cell types.

### 7. Extraction of DCs from all CD45 + cells

The count matrix is obtained from the scRNAseq data, containing gene expression levels for all CD45 + cells. The count matrix was pre-processed, which includes normalization and quality control, to remove low-quality cells and genes, ensuring robust downstream analysis. Next, the MetaCell analysis is initialized using the count matrix using R (Version 2024.04.0 + 735 (2024.04.0 + 735)). MetaCell clusters cells based on their gene expression profiles by constructing a k-nearest neighbor (k-NN) graph of cells using highly variable genes. MetaCell then partitions the k-NN graph into smaller, highly connected subgraphs called metacells.

Following this, the metacells are examined for the expression of DC markers. Specifically, the focus is on cells expressing LYZ (Lysozyme) and IRF8 (Interferon Regulatory Factor 8), which are characteristic markers for DCs. Finally, cells from the identified metacells that show high expression of Lyz and Irf8 are extracted. These extracted cells are further analyzed to study specific dendritic cell subpopulations within the CD45 + cell population.

### 8. Data Preparation and Quality Control

To analyze DCs extracted from CD45 + cells, the count matrix and metadata containing the stages of the menstrual cycle were loaded into R. The data dimensions were checked to ensure correct loading. Essential R packages, including Seurat (5.1.0, RRID:SCR_016341) (version and package identifier), tidyverse, ggpubr, Matrix, dplyr, and patchwork, were installed and loaded. A Seurat object was created using the counts data, metadata, and appropriate filtering criteria (min.cells = 3, min.features = 200). Quality control steps involved calculating the median UMI counts per cell (1719) and the median number of genes per cell (1065). The percentage of mitochondrial genes was calculated, although no mitochondrial genes were found in this dataset. Histograms of UMI counts and gene counts per cell were plotted to visualize data distribution. A violin plot of the QC columns was generated to assess the distribution of these metrics. Scatter plots comparing UMI counts to gene counts were created to detect potential outliers (correlation co-efficiency = 0.98). To ensure data quality, we filtered genes with fewer than three counts across all cells and set the clustering resolution at 0.5 to capture distinct cell populations. Differential expression analysis employed a minimum log fold change of 0.6 and an adjusted p-value threshold of 0.05.

### 9. Data Normalization and PCA

The count data were log-normalized and variable features were identified. The data were scaled and subjected to PCA. The PCA results were visualized, and the loadings were examined. The optimal number of principal components for further analysis was determined using an elbow plot.

### 10. Clustering and UMAP Embedding

A nearest neighbor graph was constructed with the selected number of dimensions (12 dimensions were selected). Clustering was performed at a resolution of 0.5. UMAP embedding was carried out to visualize the clustering results in a two-dimensional space, which was then plotted.

The steps include data preparation, quality control, normalization, feature selection, PCA, clustering, and visualization, ensuring a robust and reproducible analysis pipeline.

### 11. Additional packages used in downstream analysis (Trajectory analysis and Receptor-Ligand analysis)

Slingshot algorithm was used for lineage tracing (trajectory analysis), CellChat package was used for receptor-ligand analysis, Bioconductor and Seurat packages were used for all other analyses.

### 12. CITE-seq and Flow cytometry:

Tissue processing for validations with CITE-Seq and flow cytometry were performed as published previously (77). Dendritic cells (DCs) were characterized using flow cytometry as CD45+, CD3-, CD19-, CD56-, HLA-DRhigh, CD11c + cells, in accordance with the approach outlined by Rodriguez-Garcia et al. (2017) (58). Sample preparation for single-cell antibody and RNA sequencing was carried out as outlined by Parthasarathy et al. (2023) (50). Downstream analysis of scRNA-seq data was conducted as detailed in section 9.

### 13. Multiplex immunohistochemistry (mIHC)

The endometrial biopsy was rinsed with PBS, fixed in 4% PFA at room temperature for 4–6 hours, and subsequently dehydrated overnight. The paraffin-embedded tissue was sectioned into slides with a thickness of 4 μM and subjected to multiplex immunohistochemistry using the PANO 5-plex IHC kit (Panovue, 10144100100) and Bond Polymer Refine Detection (Leica, DS9800-CN) on the automatic dyeing machine (BOND RX, Leica). The primary antibodies were used: Anti-LYZ (Sino Biology, 110207-M095), Anti-CLEC10A (abcam, ab315086), Anti-Topoisomerase IIα (CST, 12286S), Anti-CD14 (Sino Biology, 10073-R001), Anti-IRF-8 (CST, 83413S), Anti-XCR1 (CST, 44665S), Anti-Granzyme B (Sino Biology, 10345-R002), followed by incubation with horseradish peroxidase-conjugated secondary antibodies and tyramine signal amplification. After labeling all target antigens, the nuclei were counterstained with DAPI (Panovue, Beijing, China). The multispectral images were acquired using an automated slide-scanner (SLIDEVIEW VS200, Olympus) and analyzed with HALO software (Version 3.3, Indica labs).

14. Citing Data Sources: Publicly available dataset at doi: 10.1126/science.aah4573 was used for comparison.

## Supplementary Material

Supplement 1

## Data Availability

All single-cell RNA sequencing (scRNAseq) data generated in this study will be made available in the GEO database upon acceptance of this manuscript for publication.
