# Supplementary material for: Decoding Functional and Developmental Trajectories of Tissue-Resident Uterine Dendritic Cells Through Integrative Omics
Source: Res Sq. 2024 Nov 14:rs.3.rs-5424920. Preprint. [Version 1] doi: 10.21203/rs.3.rs-5424920/v1 (PMC11601813; doi:10.21203/rs.3.rs-5424920/v1)
Supplement: Supplement 1 [file NIHPPRS5424920V1-supplement-1.pdf]

Supplementary Files

This is a list of supplementary files associated with this preprint. Click to download.

- Supplementalmaterial.pdf
